# Impact of the Polymer Backbone Structure on the Separation Properties of New Stationary Phases Based on Tricyclononenes

**DOI:** 10.3390/polym14235120

**Published:** 2022-11-24

**Authors:** Anastasiia Yu. Kanatieva, Dmitry A. Alentiev, Valeria E. Shiryaeva, Alexander A. Korolev, Alexander A. Kurganov

**Affiliations:** A.V. Topchiev Institute of Petrochemical Synthesis, Russian Academy of Sciences, Chromatography Lab, 119991 Moscow, Russia

**Keywords:** tricyclonone stationary phases, polymeric stationary phase, thermodynamic properties, dispersive surface energy, specific surface energy

## Abstract

The main purpose of this paper is to compare the chromatographic properties of capillary columns prepared with polymers with different backbone structures and to demonstrate the possibility of polymer differentiation via inverse gas chromatography. With the use of addition and metathesis types of polymerization of tricyclononenes, two new stationary phases were prepared. The metathesis polymer contained double bonds in the polymeric backbone while the backbone of the addition polymer was fully saturated and relatively mobile. A comparison of the separation and adsorption properties of new phases with conventional gas chromatography (GC) stationary phases clearly indicated their non-polar characteristics. However, the difference in the polymer structure appeared to have very little effect on the stationary phase separation properties, so other parameters were used for polymer characterization. The thermodynamic parameters of the sorption of alkanes and aromatic compounds in both polymeric stationary phases were also very similar; however, the entropy of sorption for hydrocarbons with seven or more carbon atoms was different for the two polymers. An evaluation of the specific surface energy of the stationary phases also allowed us to discriminate the two stationary phases.

## 1. Introduction

The modern practice of analytical gas chromatography is essentially a technique performed using wall-coated (WCOT) or porous layer open-tubular (PLOT) capillary columns. The columns available today afford an unprecedented level of durability, inertness, and ease of use. The invention of fused silica capillary columns in 1979 [1] and continuous innovations in stationary phase chemistry resulted in the reproducible preparation of immobilized layers with control of thickness. Many of the innovations came from academic laboratories; however, virtually all innovations in current GC column technology take place in manufacturing facilities and are indicated as proprietary information [2,3].

General information on GC columns can be found elsewhere in the literature [4], and according to [5], there are two main approaches to create PLOT capillary columns. One of them, the suspension approach [6], is based on introducing a previously prepared sorbent in the form of a suspension into a capillary with its fixation on the inner surface of the capillary. Pre-synthesized sorbents are commonly characterized by conventional analytical techniques such as porosimetry, thermogravimetry, etc. These approaches can be used for the preparation of PLOT capillary columns with both organic and inorganic sorbents. Prepared columns usually have thick layers of stationary phases (reported values are up to 50 μm) and slightly reduced efficiency compared to WCOT columns. The main drawback of PLOT columns prepared by the suspension technique is the leaching of sorbent particles from the column. This phenomenon results in signal spikes on the chromatogram and is strongly undesirable. Manufacturers offer special trap columns (e.g., PLOT Column Particle Trap by RESTEK LLC) to exclude particle spiking.

An alternative technique for the preparation of PLOT columns is the synthesis of the stationary phase directly inside the quartz capillary [5,7]. The amount of the stationary phase inside the capillary column is very small and usually cannot be characterized by the same techniques used for the characterization of the pre-synthesized sorbents. As a result, a priori, the porous properties of the prepared stationary phase remain unknown until they are investigated by chromatographic techniques. The cross-linked polymer that is formed during the synthesis is deposited onto the capillary wall and is retained due to adhesion [5,8,9,10]. Scanning Electron Microscopy (SEM) images commonly demonstrate an irregular distribution of spherical polymer particles across the column wall surface (see e.g., [10]).

At the beginning of the century, Berezkin et al. [11,12] described a new type of stationary phases for GC based on microporous membrane polymers. The most remarkable feature of membrane polymers is their ability to spontaneously form a microporous structure [13]. The other advantage of membrane polymers is the possibility of utilization of the same technique, which is conventionally used for liquid stationary phases for the formation of the stationary phase layer in the quartz capillary. Nevertheless, until now, only one membrane polymer, poly-(1-trimethylsilyl-1-propyne) (PTMSP), has been investigated in GC as a microporous stationary phase [10,11,12,14,15] for the separation of light hydrocarbons [15] and hydrides [16]. The reason for this fact is the drawback of the PTMSP-based stationary phase connected to the so-called physical aging [14], resulting in a decrease in the gas permeability of the polymer layer with time. This property of the polymers at temperatures above the glass transition temperature T_g_ is widely reported in the literature [17,18] and is interpreted using the free volume model [19]. All microporous membrane polymers have a bulky molecular structure that does not allow the formation of a densely packed structure in a solid state. With time, polymer chains tend to relax to the equilibrium state, resulting in the reduction of the polymer free volume and permeability.

Recently, a series of new membrane polymers based on norbornene monomers was synthesized and tested for membrane gas separation [20]. Some of these polymers demonstrated promising properties in the separation of light alkanes and are of interest for testing as potential stationary phases for capillary GC. Polymers used in the present work were prepared with two different mechanisms of polymerization, so they are characterized with different backbone structures and allow an elucidation of the effect of the backbone structure on the separation properties of the stationary phase. Addition polymers have relatively flexible backbones while backbones of metathesis polymers contain double bonds, preventing the free rotation of the chain. It is reasonable to expect that different rigidity levels of the polymer chains influence the selectivity and retentivity of solutes in GC separations. The energetic differences between polymeric stationary phases are elucidated by measuring the energy of the solute/polymer interactions and the thermodynamic parameters of sorption such as enthalpy, entropy or Gibbs energy. The main purpose of this paper was to compare the chromatographic properties of capillary columns prepared with polymers with different backbone structures and to demonstrate the possibility of polymer differentiation via inverse gas chromatography.

## 2. Materials and Methods

### 2.1. Chemicals

All reagents used in the study were of pure chemical grade and were purchased from Sigma–Aldrich LLC (St. Louis, MO, USA) and used without additional purification. Helium (grade A) was used as the carrier gas and was purchased from a local supplier (Dia-m, Moscow, Russia). Helium was additionally dried to remove water by passing it through a molecular sieve cartridge.

### 2.2. Synthesis of Polymers

**Figure 1:**
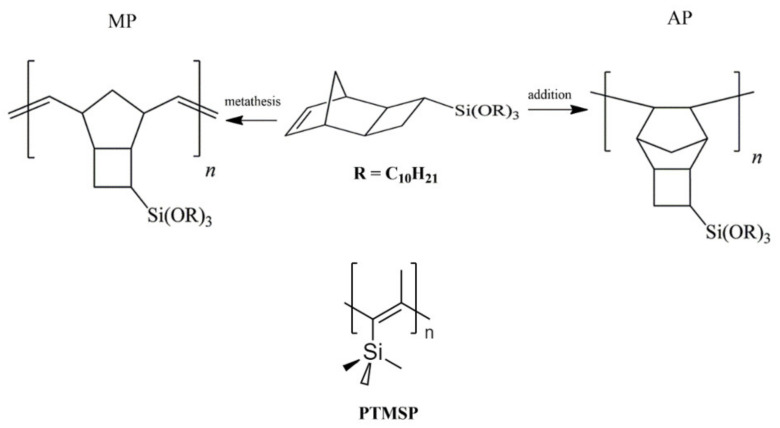
Structures of the investigated polymers together with the routes of polymerization of tricyclonones.

Addition polymer (AP) was synthesized by the polymerization of a tricyclononene monomer in the presence of a palladium-containing catalytic system, and the metathesis polymer (MP) was prepared using a niobium catalyst as described in [21]. The structures of the monomer units of the resulting polymers are shown in Figure 1, and the molecular mass characteristics of the polymers are listed in Table 1.

### 2.3. Column Preparation

The capillary columns were prepared via the static coating method. The characteristics of the prepared columns are shown in Table 1. The stationary phase layer thickness d_f_ and phase ratio β values for the prepared columns were calculated according to the known ratios [24] and are presented in Table 1.

(1) $$d_{f}=\frac{r_{c}}{2}\times \frac{C_{L}\rho }{100-\left(\frac{C_{L}}{\rho }\right)}$$

(2) $$\beta =\frac{r_{C}}{2d_{f}}$$

Here, r_c_ is the inner capillary radii; C_L_ is the concentration of the polymer in the working solution (% g/mL).

### 2.4. Chromatographic Measurements

All gas chromatographic measurements were performed on a gas chromatograph (GC 2010; Shimadzu LLC, Kyoto, Japan). The split ratio was 1:50, and detection was performed by means of a flame ionization detector (FID). Inverse Gas Chromatography (IGC) measurements were carried out within the temperature range of 40 to 100 °C except for the PTMSP column, where the working interval was 70–150 °C. The test mixture containing 12 components—n-alkanes and aromatic compounds—was prepared from individual substances. Data were acquired using the software package GC-solution (Shimadzu LLC, Kyoto, Japan) and were evaluated using Origin software (version 2018, OriginLab Corporation, MA, USA).

### 2.5. Thermogravimetric and Calorimetric Measurements

Thermogravimetric analysis (TGA) was carried out using a Perkin–Elmer TGA-7 (Artisan Technology Group, Champaign, IL, USA) instrument. Calorimetric measurements were conducted using a differential scanning calorimeter (“Mettler” TA-4000) at a heating rate of 20 °C/min under an argon atmosphere.

### 2.6. Testing Columns for Physical Ageing

To evaluate the thermal stability of the stationary phases based on AP and MP, the column was heated to a given temperature for 1 h and then cooled to 40 °C [25]. The test mixture of n-alkanes and aromatic compounds was then separated under standard conditions (40 °C, 70 kPa). Then, the column heating procedure was repeated with a heating temperature step of 20 °C. The thermal stability of the column was studied in the temperature range of 40–200 °C. The retention factor k and column efficiency N (number of theoretical plates) were calculated for each analyte at each temperature.

## 3. Results and Discussion

Taking into consideration all of the acquired data, we now discuss the influence of the backbone structure of the polymer on the thermal stability of the prepared GC columns, their chromatographic properties such as retention parameters and column selectivity, and the thermodynamic parameters of the sorption of hydrocarbons of different classes on the addition and metathesis polymers.

### 3.1. Synthesis and Thermal Stability of Polytricyclononenes

Tricyclononene and its derivatives can be polymerized via the addition type of polymerization to the addition polymer (AP) or via ring opening metathesis polymerization to the metathesis polymer (MP) (Figure 1) [21]. The backbone of MP polymers contains double bonds that impede the rotation of the chain segments. Correspondingly, the glass transition temperature (T_g_) of MP is expected to be higher than that of AP, which is characterized by the relatively flexible saturated carbon main chain [21]. The difference in glass transition temperatures (T_g_ values) between MP and AP was rather small: −63 °C for AP and −71 °C for MP. However, both T_g_ values were well below the conventional temperature range used in GC. This fact ensured the viscoelastic state of both polymers at gas chromatographic separation temperatures.

**Figure 2:**
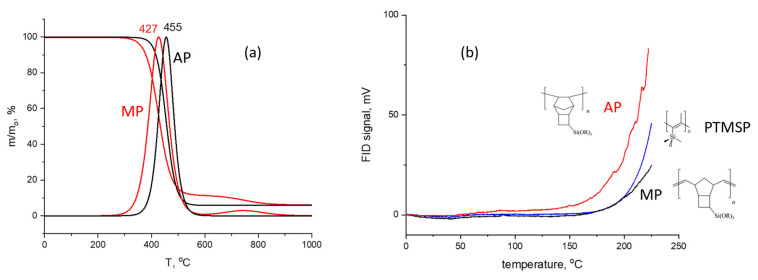
TGA curves demonstrating the percentage mass loss of the polymers depending on the temperature (**a**) and baseline stability under GC conditions of the AP and MP stationary phases and the PTMSP stationary phase (**b**).

The thermostability of the new stationary phases was tested by TGA (Figure 2a) and by a chromatographic method by monitoring the base lines for both columns with a gradual increase in the column temperature (Figure 2b). The TGA curves were conventional for the organic polymers’ S-shaped profile. The temperature at which the polymer lost 5% of its mass was 362 °C for MP and 400 °C for AP, correspondingly. Speed of polymers decomposition reached maximum at 427 °C for the MP polymer and 455 °C for the AP polymers. Therefore, thermogravimetric measurements demonstrated a slightly higher thermal stability of the AP polymer over its MP counterpart. However, the baseline drift for the column based on the MP stationary phase under temperature programming was lower than that for the AP-based column (Figure 2b). The baseline level for the column with the MP stationary phase remained stable up to 225 °C. The AP-based stationary phase demonstrated a significant increase in the baseline signal at 175 °C. For comparison, we also measured the baseline drift for the PTMSP stationary phase, as PTMSP had an unsaturated carbon backbone similar to that of MP (Figure 1). As one can see from Figure 2, the baseline drift for the PTMSP stationary phase was similar to that of the MP-based column (Figure 2b). Thus, the data obtained via the chromatographic measurements were in contradiction with the data obtained by TGA, likely due to the thermal stabilizing effect of the double bonds in the polymeric backbone. The reason for this effect might be the much higher sensitivity of the IGC method compared to TGA: the baseline started to increase with minor bleeding of the stationary phase from the column.

Both new stationary phases were tested for physical ageing as described in the Materials and Methods. However, no changes in selectivity and efficiency were observed when the columns with AP and MP stationary phases were heated within a few hours at increased temperatures up to 175 °C. These data coincide well with the stability test of the AP and MP membranes used in membrane chemistry, which could not detect physical ageing of these polymers [21].

### 3.2. Chromatographic Performance of Tricyclononene Stationary Phases

It was reported in a membrane study [21] that AP and MP were different in their gas permeability properties [21,26], and the permeability of MP was higher than that of AP. It was also demonstrated that both polymers are capable of membrane separation of hydrocarbons [26].

**Figure 3:**
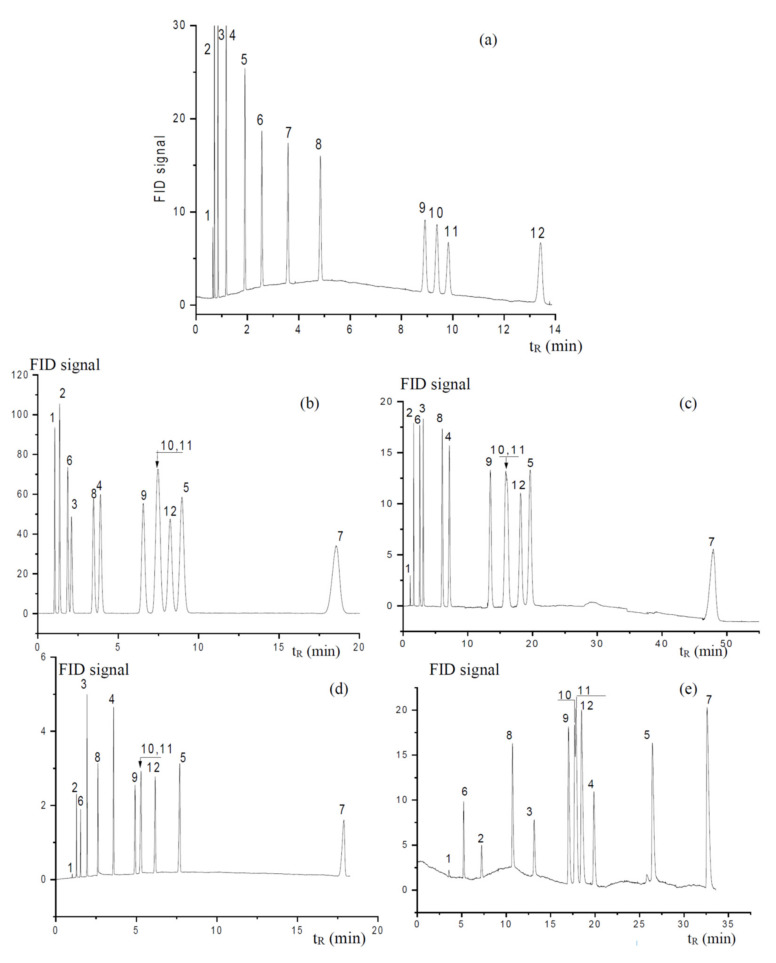
Separation of the test mixture during the investigated stationary phases: (**a**)—PEG 20 M, (**b**)—AP, (**c**)—MP, (**d**)—SE-30, (**e**)—PTMSP. Test mixture: 1—n-pentane, 2—n-hexane, 3—n-heptane, 4—n-octane, 5—n-nonane, 6—benzene, 7—n-decan, 8—toluene, 9—ethylbenzene, 10—p-xylene, 11—m-xylene, 12—o-xylene. Separation conditions were as follows: column temperature 40 °C, isothermal for (**a**–**d**) chromatograms, and 130 °C for (**e**) chromatogram, carrier gas helium, inlet pressure 70 kPa.

Figure 3 demonstrates the separation of the test mixture of twelve components—n-alkanes and aromatic hydrocarbons– in the AP and MP stationary phases together with three conventional stationary phases: non-polar stationary phase polydimethylsiloxane SE-30, polar polyethylene glycol stationary phase PEG 20M, and the microporous polymer polytrimethylsilylpropyne PTMSP stationary phase. As expected based on the properties of the presented stationary phases, polar stationary phase PEG 20M weakly retained n-alkanes, while the fairly polarizable aromatic compound had the highest retention times (Figure 3a). The PEG 20M stationary phase also ensured the good separation of the critical triad (ethylbenzene/m-xylene/p-xylene), and aromatic solutes far behind the n-alkanes with an equal number of carbon atoms, e.g., benzene (six C atoms), were eluted between n-nonane and n-decan, and toluene (seven C atoms) was eluted after n-decan. The elution order of the test solutes in the PEG 20M stationary phase also differed from that for the non-polar stationary phase SE-30 (Figure 3d): benzene was eluted between n-hexane and n-heptane, the toluene retention time was between that of n-heptane and n-octane, and the last eluted solute was n-decan. It is known that the non-polar stationary phase usually cannot separate the critical ethylbenzene/m-xylene/p-xylene triad.

The elution order for the test mixture during the AP and MP stationary phases was similar to the elution order for the non-polar stationary phase (Figure 3b–c): the last eluted sorbate was n-decan, benzene was eluted between n-hexane and n-heptane, and the toluene peak was situated between n-heptane and n-octane. It was also found for the tricyclonone stationary phases that they could not separate the critical triad. Nevertheless, there were also obvious differences in the sorbate elution order for the tricyclononene and dimethylsiloxane stationary phases (Figure 3b–d). Aromatic compounds were eluted during the non-polar stationary phase SE-30 close to the corresponding n-alkane. As for the AP and MP stationary phases, hydrocarbons with the aromatic rings were eluted close to the next n-alkane. This difference might be caused by the presence of microporosity in the AP and MP stationary phases. The effect of microporosity might be confirmed by comparison of the separations performed during the AP and MP stationary phases with the separations performed during the PTMSP stationary phase (Figure 3e). PTMSP is a microporous polymer with a glass transition temperature lower than the working temperatures of the GC method. This polymer is characterized by a high free volume and has a developed surface area [23] available to the sorbates ensuring the chromatographic process. As shown in Figure 3, the remarkable feature of the microporous stationary phases was the elution of xylenes and ethylbenzene as a joint group. The same phenomenon was also observed for the AP and MP stationary phases. However, the retention of the PTMSP phase was significantly higher than that of the AP- or MP-based capillary columns. The test mixture could not be separated on the PTMSP column isothermally at 40 °C due to the extremely high retention times of the analytes, and the temperature had to be increased to 130 °C.

The polarity of the stationary phase is also a very important parameter for chromatographic separations, and it is frequently characterized by McReynolds constants, which are evaluated from the retention times of five probe analytes: benzene, 1-butanol, 2-pentanone, 1-nitropropane, and pyridine [27]. Both AP and MP were tested using McReynolds analytes, and the calculated average polarity of the AP and MP stationary phases was 175 and 186, respectively, indicating the low polarity of these stationary phases. The low polarity of the stationary phases was probably connected to the absence of polar groups in the polymer structure and the dense shielding of the highly polyrizable double bonds in the carbon bone of the MP polymer, which makes them inaccessible to the analytes during chromatographic separations. Taking into consideration all of the acquired data, the AP and MP stationary phases may be described as non-polar stationary phases in which selectivity is slightly affected by polymer microporosity. Therefore, the chromatographic retention data were found to be insufficient to discriminate between these two isomeric stationary phases, so other parameters such as the thermodynamic parameters of sorption had to be used.

### 3.3. Thermodynamic Parameters of Sorption for Separations during AP and MP Stationary Phases

The thermodynamic parameters of sorption for analyte separation in GC are usually evaluated by means of IGC [28]. The theoretical background of IGC is described in the literature in detail [29,30]. The thermodynamic parameters of sorption that are usually evaluated via IGC are the enthalpy of sorption ΔH^o^ and the entropy of sorption ΔS^o^. They are related to the partitioning coefficient of the analyte K (Henry constant) between the moving and stationary phases according to the equation

ΔG^o^ = −RTlnK = ΔH^o^ − TΔS^o^ (3)

where ΔG^o^ is the free energy of sorption; R is the universal gas constant; $K=\frac{C_{s}}{C_{m}}$ is an equilibrium constant, C_s_ and C_m_ are analyte concentrations in the stationary and mobile phases, respectively; and T is the column temperature, K. The partitioning coefficient K correlates with the retention factor k [22,29]:

K = kβ(4)

where $\beta =\frac{V_{m}}{V_{s}}$ is the phase ratio, i.e., the ratio of the mobile phase volume V_m_ to that of the stationary phase V_S_ in the column, and $k=\frac{t_{R}-t_{0}}{t_{0}}$ is the retention factor with t_R_ and t_o_ being the analyte retention time and column hold-up time, respectively. Combining Equations (3) and (4) allows one to obtain the relationship allowing the evaluation of the thermodynamic parameters of sorption from the linear correlation between lnk and 1/T:

(5) $$\mathrm{lnk}=\frac{-\Delta H^{o}}{\mathrm{RT}}+\frac{\Delta S^{o}}{R}-\ln\beta$$

**Figure 4:**
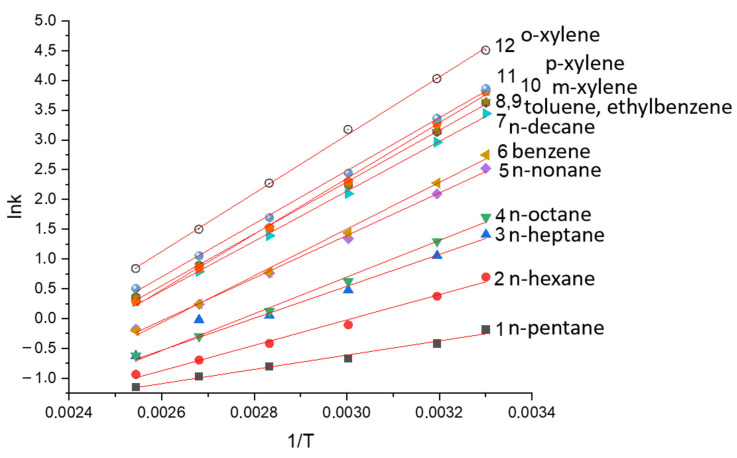
Linear dependence of lnk on inverse column temperature 1/T_c_ for solutes of the test mixture in the MP stationary phase. The designation of solutes is shown in Figure 3.

**Figure 5:**
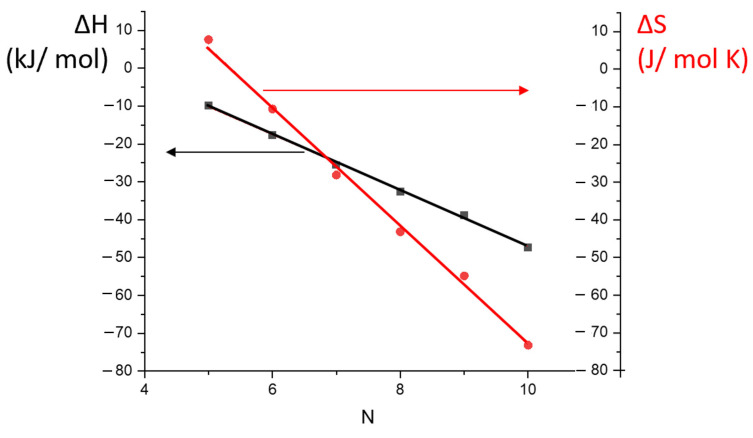
Correlation of the enthalpy ΔH and entropy ΔS of the sorption of n-alkanes during the PM stationary phase with the methylene group number for the n-alkane molecules.

Corresponding straight-line correlations were observed for all of the polymeric stationary phases studied in the research (see, e.g., Figure 4), and the calculated values of the differential enthalpy ΔH^o^ and entropy ΔS^o^ of sorption for the investigated saturated and aromatic hydrocarbons are shown in Table 2. It is usually considered that changes in the distribution of analyte molecules between the stationary and mobile phases are mainly due to the size, geometrical structure, and electronic configuration of solutes on the one hand and the structure and conformation of the polymeric stationary phase on the other hand. The AP and SE-30 stationary phases do not have any functional groups, and there was no reason to expect any specific interactions with polar analytes for these stationary phases. In contrast, the MP and PTMSP stationary phases have unsaturated structures with double bonds. Potentially, these stationary phases could result in π → π or other types of specific interactions with a polar solute due to high polarizability. However, the data presented in Table 2 do not support this assumption, and the sorption properties of AP and MP were found to be very similar. As one can see from Figure 5, both stationary phases exhibited linearity of the differential enthalpy ΔH^o^ and entropy ΔS^o^ of sorption for the n-alkane carbon number. The slopes of the linear dependences are called the methylene selectivity of the stationary phase and are shown in Table 3 together with similar characteristics of the discussed conventional GC stationary phases. The enthalpy-based methylene selectivity values of the new stationary phases and of the non-polar SE-30 were very close, supporting the non-polar nature of the AP and MP stationary phases. Enthalpy-based methylene selectivity of polar stationary phase PEG 20M was very different from the corresponding values of the AP and MP stationary phases. A similar difference was observed for the entropy-based methylene selectivity. It was also found that the difference in the values of the entropy of sorption increased with the carbon number of the analyte, and the entropy values for hydrocarbons after n-octane may be used for differentiation between the isomeric polymeric stationary phases. The higher entropy loss for the addition polymer may be connected to the higher flexibility of the carbon chain of the AP, resulting in more intense interactions of the analytes in the stationary phase.

**Figure 6:**
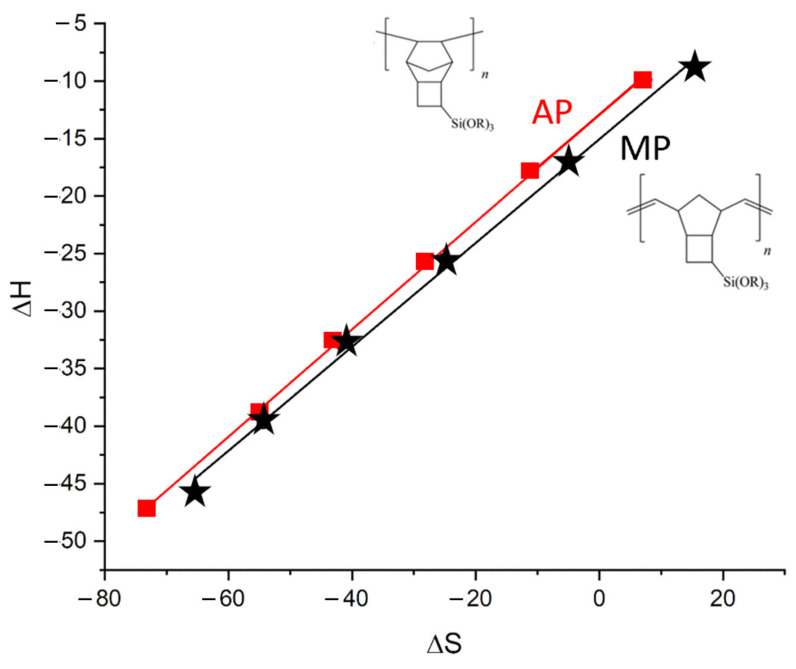
Compensation plots for the sorption of n-alkanes during the AM and PM stationary phases.

Additional similarity of the non-polar stationary phases was revealed when studying the entropy–enthalpy compensation effect. This effect is described as a linear correlation of enthalpy versus the entropy of sorption with the slope being the compensation temperature T_C_. The value of the compensation temperature is considered to be the process characteristic [31]. Chemical reactions or equilibrium processes with similar compensation temperatures are usually considered to have similar mechanisms and are called isokinetic or iso-equilibrium processes. The compensation plots for the AP and MP stationary phases are shown in Figure 6, and the compensation temperatures of the stationary phases are shown in Table 3. Surprisingly, the lowest compensation temperatures were observed for the AP and MP stationary phases and the highest for PEG 20M, which again clearly showed the non-polar characteristics of the new stationary phases.

### 3.4. Evaluation of the Surface Free Energy for the AP and MP Stationary Phases

One of the most used applications of IGC is the determination of the surface free energy [28,32,33]. Conventionally, the surface energy is described by the combination of the dispersive and specific nondispersive properties of the polymer. Dispersive properties are usually obtained from the dispersive contribution of the surface free energy. Specific properties are determined by the parameters that are connected to the surface ability to be an electron acceptor or electron donor. The sum of the dispersive and specific components represents the total solid surface energy [28].

The dispersive free energy γ_s_^D^ of the AP and MP stationary phases was evaluated by the Dorris–Gray method [34]. This method utilizes the methylene selectivity of free energy ΔG_CH2_, which is evaluated from the linear dependence of ∆G = −RTlnkβ on the carbon number of n-alkane, which is used as the test solute (Table 3, Figure 5) [28]:

(6) $$\gamma_{s}^{D}=\frac{{\left(\Delta G_{CH2}\right)}^{2}}{4\;N^{2}{\left(\alpha_{CH2}\right)}^{2}\gamma_{CH2}}$$

Here, α_CH2_ is the cross-sectional area of a CH_2_ group (m^2^) [31]: α_CH2_ = 69.919/(568.02–T)^0.5^ (Å^2^), N is the Avogadro number, 6.023 × 10^23^, γ_CH2_ is the free energy of a CH_2_ group [27]: γ_CH2_ = 35.6 + 0.058 (293–T) (mJ/m^2^).

**Table 3:** Thermodynamic parameters of the sorption of the investigated stationary phases.

Stationary Phase	Thermodynamic Parameters			Dispersive Surface Energy *)	Specific Surface Energy ∆G^SP^, kJ/mol (See Figure 7)					
	−ΔH, kJ/mol	−ΔS, J/molK	T_com_	γ_s_^D^, mJ/m^2^	Benzene	Toluene	Ethylbenzene	m-Xylene	p-Xylene	o-Xylene
AP	7.2	16.2	415	56.1	3.6	3.7	3.6	4.0	2.8	4.4
MP	7.4	15.7	467	57.7	3.7	3.7	3.4	3.6	2.5	4.4
PEG20M	3.5	3.9	910	97.1	9.6	9.0	8.4	8.4	7.0	9.4
PTMSP	7.2	12.5	509	49.3 **	3.4	2.8	2.4	3.0	0.6	2.2
SE-30	7.3	10.9	486	48.2	2.6	2.5	2.5	3.2	1.6	2.5

*) at 40 °C; **) at 50 °C.

As one can see from Table 3, the AP- and MP-based stationary phases demonstrated almost identical values of the dispersive free energy γ_s_^D^ and, correspondingly, had an equal tendency for non-specific interactions with solutes. These values were 1.5 times higher than the free dispersive energy of the polar PG 20M stationary phase. Low values for PEG 20M can be explained by weak interactions of n-alkanes (used as solutes) with the polar surface PEG 20M. The small increase in the free surface energy of AP and MP over SE-30 and PTMSP may be attributed to the non-polar features of the polymer structure.

**Figure 7:**
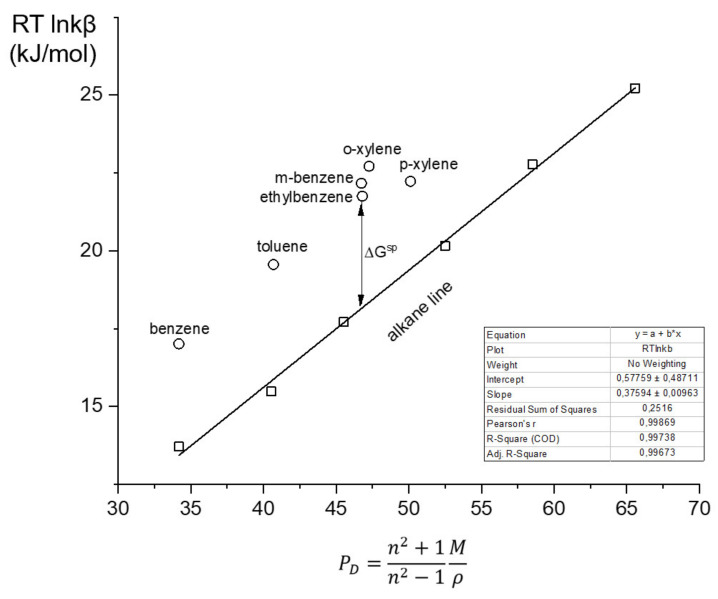
Diagram illustrating the evaluation of the specific free energy (ΔG^SP^) by the polarization method.

The specific surface energy of the stationary phases was evaluated based on interactions with polarized analytes, which were aromatic hydrocarbons in this study. The carbon backbone of the MP-based stationary phase provided a high number of double bonds (Figure 1), which could ensure π–π interactions with the aromatic rings of the analytes and indicate a difference with the AP stationary phase. Commonly, when the Dorris–Gray concept is used for the evaluation of the dispersive free energy, the specific contribution is calculated using the polarization method [28]. As an intrinsic characteristic, independent of the test analyte nature, which is used in IGC to receive the data for the polarization method, the value of the deformation polarization (P_D_, cm^3^/mol) is used:

(7) $$P_{D}=\frac{n^{2}+1}{n^{2}-1}\frac{M}{\rho }$$

where n, M, and ρ are the respective refractive index, molar mass (g/mol), and density (g/cm^3^) of the test molecule. In the first step, the graphical dependence of the free energy of adsorption on ∆G = −RTlnkβ on the molar deformation polarization P_D_ for n-alkanes was plotted. The obtained straight-line dependence is called the alkane line (Figure 7) and is connected to the dispersive interaction energy [35]. In the next step, points of other solutes were plotted in the graph, and the vertical distance between polarized analyte points and the alkane line yielded the specific free energy (ΔG^SP^) (Figure 7). The values of the specific free energy ΔG^SP^ are shown in Table 3 [35]. The specific free energy of the MP stationary phase was higher than the corresponding value for the AP stationary phase. However, the differences were rather small and close to the accuracy level. It is likely that the interaction of aromatic rings and double bonds is prohibited by the steric surrounding of the double bonds. The existence of steric hindrances for solute adsorption during the investigated stationary phases may be deduced from the DG^SP^ values (3–4 kJ/m^2^), which are two to three times lower than those values, e.g., for the adsorption of dichloromethane on the quartz surface (11.5 kJ/m^2^ [28]). These obstacles probably do not exist for sorption onto the PEG 20M surface where DG^SP^ values (7.5–9.5 kJ/m^2^) were quite close to the literature values.

## 4. Conclusions

New representatives of microporous polymeric matrixes, addition and metathesis polymers of tricyclononene, were synthesized using different catalytic systems and were investigated with the IGC method to differentiate the isomeric stationary phases using chromatographic data. The backbone of the metathesis polymer had numerous double bonds while the backbone of the addition polymer was saturated. The presence of double bonds made the MP polymeric chains rigid and conformationally less flexible. In contrast, the addition AP polymeric chains seemed to be more mobile. However, the difference in the polymer carbon backbone structures had very little effect on the separation properties of the stationary phase. The elution sequence of the test solutes and McReynolds coefficients were similar for both stationary phases prepared from AP and NP polymers. The evaluation of the thermodynamic parameters of sorption such as the enthalpy and entropy of the sorption of alkanes and aromatic compounds, methylene selectivity, and the compensation temperatures did not reveal significant differences between the stationary phases. The only parameter that was able to differentiate the isomeric polymeric stationary phases was the entropy of sorption for hydrocarbons with nine or more carbon atoms. The evaluation of the specific surface energy for aromatic analytes also allowed the discrimination of the polymers. This high similarity between the AP and MP polymers points to the high-shielding double bonds in the MP polymer in contradiction to the clear difference observed between the polymers and the typical polar stationary phases.

## Figures and Tables

**Table 1:** Physical properties of GC columns and stationary phases used in the study.

Column	Stationary Phase	Molar Mass/Polydispersity, kDa	Glass Transition Temperature T_g_, °C	Decomposition Temperature T_c_, °C	Column Length L, m	Column Inner Diameter d_c_, mm	Stationary Phase Thickness d_f_, μm	Phase Ratio β
1	AP	600/3.2	−63 [21]	400 **	10.0	0.165	0.25	165
2	MP	1000/2.5	−71 [21]	360 **	10.0	0.165	0.25	165
3	SE-30	1500/2.5	−120 [22]	>350	15.0	0.23	0.22	229
4	PEG 20M	20/1.05	−52/+50 * [22]	290 ** [22]	16.0	0.165	0.25	165
5	PTMSP	200/1.2	>300 [23]	>350 [23]	15.0	0.165	0.25	165

*) Glass transition point/melting point; **) Temperature at which 5% weight loss occurred.

**Table 2:** Thermodynamic parameters of the adsorption of test solutes during the investigated polymeric stationary phases (r, s.d. 10%) *.

Polymer	AP		MP		SE-30		PEG 20M		PTMSP	
Solute	ΔH, kJ/mol	ΔS, J/molK	ΔH, kJ/mol	ΔS, J/molK	ΔH, kJ/mol	ΔS, J/molK	ΔH, kJ/mol	ΔS, J/molK	ΔH, kJ/mol	ΔS, J/molK
C5	−8.9	15.5	−9.9	7.53	−	−	−17.6	−42.16	−45.6	−78.5
C6	−17.1	−5.0	−17.7	−10.76	−27.1	−48.1	−21.6	−45.23	−48.2	−74.3
Bzl	−21.4	−13.9	−22.3	−19.65	−	−	−30.4	−50.0	−51.7	−86.0
C7	−25.5	−25.0	−25.6	−28.22	−30.3	−50.6	−25.4	−50.2	−55.4	−88.5
Tol	−29.7	−32.3	−29.8	−34.87	−	−	−33.4	−53.1	−56.4	−89.3
C8	−32.8	−41.9	−32.6	−43.18	−33.8	−54.3	−29.4	−53.8	−60.4	−93.5
EtBzl	−35.3	−43.3	−34.9	−48.59	−	−	−36.7	−58.4	−60.8	−94.3
m-Xyl	−36.6	−46.1	−36.0	−46.51	−	−	−36.2	−56.2	−59.7	−95.1
p-Xyl	−36.9	−46.8	−36.2	−47.34	−	−	−37.1	−58.6	−60.8	−93.5
o-Xyl	−37.7	−46.9	−38.9	−54.82	−	−	−37.4	−57.2	−60.3	−91.8
C9	−39.7	−54.3	−37.1	−48.17	−37.6	−59.0	−32.0	−57.7	−70.8	−110.9
C10	−45.7	−65.3	−40.7	−53.99	−41.5	−63.9	−35.3	−61.0	−75.8	−116.7

*) temperature range is 50–95 °C except for PTMSP where the temperature range is 70–150 °C.
